# Risk factors associated with the loss of cartilage volume on weight-bearing areas in knee osteoarthritis patients assessed by quantitative magnetic resonance imaging: a longitudinal study

**DOI:** 10.1186/ar2272

**Published:** 2007-07-31

**Authors:** Jean-Pierre Pelletier, Jean-Pierre Raynauld, Marie-Josée Berthiaume, François Abram, Denis Choquette, Boulos Haraoui, John F Beary, Gary A Cline, Joan M Meyer, Johanne Martel-Pelletier

**Affiliations:** 1 Osteoarthritis Research Unit, University of Montreal Hospital Center, 1560 Sherbrooke Street East, Montreal, QC, Canada H2L 4M1; 2 Radiology Department, Maisonneuve-Rosemont Hospital, 5415, boulevard de l'Assomption, Montreal, QC, Canada H1T 2M4; 3 Research & Development, ArthroVision, 1871 Sherbrooke Street East, Montreal, QC, Canada H2K 1B6; 4 Health Care Research Center, Procter & Gamble Pharmaceuticals, 8700 Mason-Montgomery Road, Mason, OH 45040-9462, USA

## Abstract

The objective of this study was to identify, on a symptomatic knee osteoarthritis (OA) cohort, the risk factors associated with the progression of the disease. More specifically, we investigated the correlation between knee cartilage volume loss from subregions over the span of 24 months by means of quantitative magnetic resonance imaging (qMRI) with demographic, clinical, radiological, and MRI structural changes.

A cohort of 107 patients with knee OA selected from a large trial evaluating the effect of a bisphosphonate underwent x-rays and MRI of the knee at baseline and 24 months. Joint space width (JSW) and joint space narrowing (JSN) and cartilage volume loss over time in subregions of the tibial plateaus and femoral condyles were quantitated. Structural changes in the subchondral bone (hypersignal) and in the menisci (tear and extrusion) were also evaluated.

The greatest cartilage volume loss was found in the medial compartment, and risk factors included female gender, JSW, meniscal lesions, and bone changes at baseline. Subregion analysis revealed that the greatest cartilage volume loss at 24 months was found in the central area of the medial tibial plateau (15%; *p *< 0.0001) and of the medial femoral condyle (12%; *p *< 0.0001). These findings were associated with the presence at baseline of meniscal extrusion, particularly severe meniscal extrusion, medial and severe meniscal tear, bone hypersignal, high body mass index (BMI), smaller JSW, increases in Western Ontario and McMaster Universities Osteoarthritis Index (WOMAC) pain and patient global scores over time, and greater JSN. Parameters predicting medial central femoral condyle cartilage volume loss at 24 months were lateral meniscal tear, SF-36 and BMI at baseline, and JSN. At the medial central tibial plateau, the parameters were severe meniscal extrusion, severe lateral meniscal tear, and bone hypersignal in the lateral compartment at baseline, and WOMAC pain change.

Meniscal damage and bone changes are the features most closely associated with the greatest subregional cartilage volume loss. Interestingly, for the first time, JSN was strongly associated with cartilage loss in the central areas of plateaus and condyles. This study also further confirms the correlation between cartilage volume loss and JSN and symptomatic changes at 24 months.

## Introduction

The structural changes in knee osteoarthritis (OA) are characterized mainly by the progressive erosion and loss of articular cartilage [1]. These changes are often associated with additional structural changes such as subchondral bone lesions, which include remodelling and cysts, and alterations in the menisci, which include degeneration, tear, and extrusion [2,3]. Conventional x-rays have been used and continue to be used to assess some of these changes, particularly in the evaluation of disease progression. However, the use of x-rays to assess and quantify structural changes over time does present some serious limitations, including the fact that this technology does not permit direct visualization of cartilage) [4-7].

In the last decade, remarkable progress in the development of imaging technology has been made. Magnetic resonance imaging (MRI) now allows not only the direct visualization of joint structure but also the quantitative assessment of changes over time. A number of semiquantitative scoring systems and quantitative technologies have been developed to achieve this goal [8]. Most of the work has concentrated on the measurement of cartilage volume/thickness and the assessment of changes to evaluate the evolution of OA lesions in cross-sectional and longitudinal studies. Some of these studies have been highly instrumental in providing a significant amount of new information. For instance, they have shown that disease progression is not consistent among patients suffering from knee OA and that a number of factors are associated with a risk of more aggressive progression. These risk factors include higher body mass index (BMI), meniscal tear/extrusion, and subchondral bone marrow hypersignal or edema [2,5,7,9-11]. To date, correlations between the global or regional loss of cartilage and disease symptoms or patient function have seldom been studied and correlations between x-ray and MRI data with respect to cartilage loss are recognized in general as not very strong) [4-7]. Studies using quantitative MRI (qMRI) have demonstrated that the loss of cartilage volume in patients with knee OA is generally progressive over time and is usually greater in the medial compartment than the lateral compartment [2,4,7,11,12]. However, in these patients, very little information is available on the evolution of cartilage loss over time in the more focal regions, such as the subregions of the knee compartments, and on whether patients with rapid versus slow disease progression have the same evolution. Even less information is available on the relationship between these lesions and associated risk factors, disease signs and symptoms, and x-ray changes. Therefore, the main aim of this study was to identify the structural changes in OA, which could explain the progression of symptoms, and thereby provide a better understanding of the natural evolution of the disease. This information is essential to the design of clinical trials and the development of new therapeutic disease-modifying OA drug (DMOAD) strategies.

## Materials and methods

### Patient selection

A subset of 110 patients (107 completed the study) was selected from 1,232 patients from North America enrolled in a large clinical trial evaluating the impact of risedronate, a bisphosphonate, on knee OA as previously described [13]. In this latter study, the patients were randomly assigned equally into four treatment groups: placebo, risedronate 5 mg/day, risedronate 15 mg/day, or risedronate 50 mg/week. The patients [13] had symptomatic disease that required medical treatment in the form of acetaminophen, traditional nonsteroidal anti-inflammatory drugs (NSAIDs), or selective cyclooxygenase-2 inhibitors. Eligible patients were required to display radiological evidence of OA of the affected knee on a radiograph obtained within 6 months of the outset of the study. Finally, patients had to have a minimum joint space width (JSW) of the medial compartment of between 2 and 4 mm, at least one osteophyte, and a narrower medial compartment compared with the lateral compartment. The measurements were done from a baseline film using the standardized semiflexed view, which was contrasted with follow-up films [7,13]. No patient had sole lateral compartment disease.

Patients were excluded if they had chondrocalcinosis or an acute or chronic infection (including tuberculosis) or if their OA of the knee was secondary to other conditions. Further exclusion factors included history of past or present gastrointestinal ulceration, receipt of an intra-articular corticoid injection in the study knee within the 6 months prior to the outset of the study, as well as classification as radiological grade IV on the Kellgren-Lawrence scale for the study knee or severe (class IV) functional disability. In the case of patients with two symptomatic knees, the more symptomatic knee was chosen for the investigation. Patients were permitted to receive simple analgesics or NSAIDs, with the exception of indomethacin [14], the regimens of which could be changed according to the preference of the rheumatologist and the clinical course of the patient. Such regimens, as well as any changes to them, were closely monitored and noted. A centralized ethics committee approved this study, and each patient gave informed consent.

### Clinical evaluation

Patients underwent clinical evaluation at baseline and every 6 months thereafter until 24 months. They were first evaluated on the basis of the Western Ontario and McMaster Universities Osteoarthritis Index (WOMAC) [15], using its French-Canadian translation [16]. In addition, the patients themselves used a visual analog scale to make a global assessment of their condition (patient global assessment: 0 = very good; 100 = very bad) and to rate the pain they were experiencing that day (patient pain score: 0 = no pain; 100 = most severe pain). Finally, the SF-36, a generic quality-of-life instrument, was administered to the patients at each visit [17]. A washout of medications was done prior to the clinical evaluation; NSAIDs were discontinued at least 48 hours prior to the investigation and acetaminophen, 24 hours. The clinical evaluators were blinded to the results of previous radiological or MRI data.

### Knee x-rays

The JSW of the target knees at baseline and 24 months of follow-up, at the narrowest point in the medial tibio-femoral compartment, was measured according to the published protocol by means of an automated computerized method of measurement [18,19]. In the rare occurrence that the radiographic quality of the film prevented the implementation of automatic JSW measurement software, manual intervention was required [20]. The variation coefficient for JSW measurement for the original reliability study was 1% for repeat radiographs (test/retest) of the knee in the semiflexed position [18]. The reproducibility of the method was also reassessed [21]; data showed that 45% of the examinations achieved high quality (that is, JSW difference between repeat films of less than 0.1 mm) and 92% achieved excellent to good quality with a difference between repeat films of 0.3 mm.

### Knee magnetic resonance imaging

High-resolution, three-dimensional MRI for each patient with OA (baseline and 24 months) was acquired using the commercially available Magnetom Vision 1.5 Tesla machine with integrated knee coil (Siemens, Erlangen, Germany) as previously described [22,23]. These exams are optimized three-dimensional fast inflow with steady-state precession (FISP) acquisitions with fat suppression. This registration procedure previously demonstrated excellent intra- and inter-reader correlations [23].

**Figure 1:**
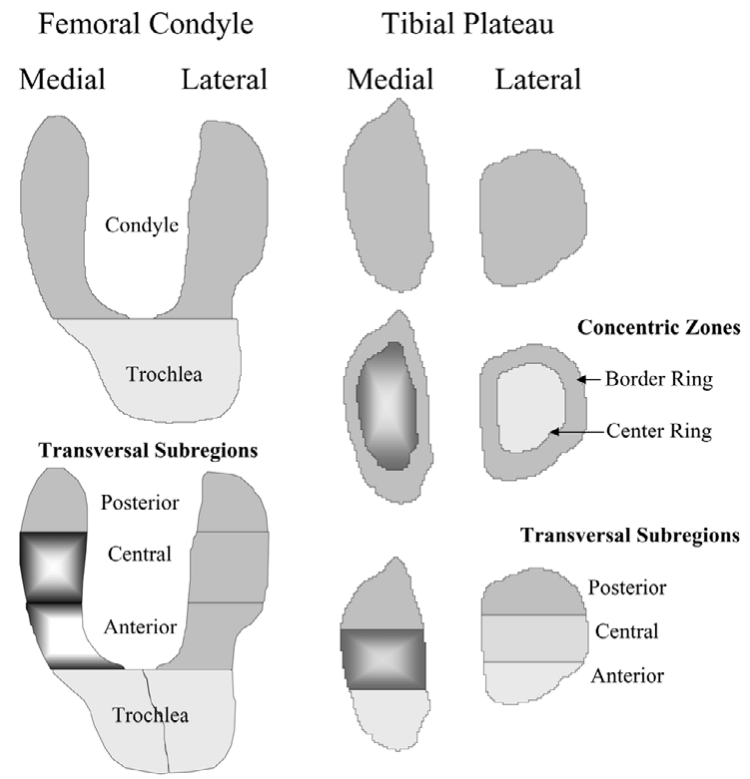
Graphic representation of the cartilage subregions as described in Table 2. Of note is that the greatest cartilage volume loss at 24 months was found for the femoral condyle at the medial central and anterior regions and for the tibial plateau at the medial center ring and central region (shading from center).

The cartilage volume (cubic millimeters) was calculated [22,23] and change in cartilage volume over time was calculated compared with baseline in absolute values (cubic millimeters) and expressed as a percentage as previously described [22]. The cartilage volume was evaluated in different regions by means of the WORMS (whole-organ magnetic resonance imaging score) system [24] with slight modification (Figure 1). The femoral articular and trochlear surfaces were divided into medial and lateral regions. The medial and lateral femurs were each divided into three regions: anterior, central, and posterior. The medial tibial plateau and lateral tibial plateau were each divided into three equal regions (anterior, central, and posterior) or in concentric zones: border and center rings.

### Meniscal and bone lesions

The evaluation of meniscal and bone structure was performed using the same sequences as those used for the cartilage assessment [2]. The FISP sequence enabled visualization of the meniscal tissue and bone lesions with enough clarity to adequately and reliably perform the semiquantitative scoring system. A semiquantitative assessment of meniscal lesions and bone hypersignal (edema) was performed by an experienced radiologist (M-JB) who was blinded to the time sequences and cartilage volumes.

The scoring system [2,7] for meniscal damage referred to the accepted MRI nomenclature for meniscal anatomy [25,26]. In brief, the section of the menisci affected by tear or extrusion was scored separately using the semiquantitative scales [2]. Meniscal tear assessment was as follows: 0 = no damage, 1 = 1 out of 3 meniscal areas involved (anterior, middle, and posterior horns), 2 = 2 out of 3 involved, and 3 = all 3 areas involved (severe tear). The extent of meniscal extrusion on the medial or lateral edges of the femoro-tibial joint space, not including the osteophytes, was evaluated for the anterior, middle, and posterior horns of the menisci in which 0 = no extrusion, 1 = partial meniscal extrusion, and 2 = complete meniscal extrusion with no contact with the joint space (severe extrusion).

For bone hypersignal, the extent of the lesion was assessed in the medial and lateral tibio-femoral compartments (as previously described [7]) with the following semiquantitative scale: 0 = absence of hypersignal; 1 = mild to moderate hypersignal (a small- or medium-sized lesion); and 2 = severe hypersignal (a large-sized lesion). The results are presented by either absence or presence of any hypersignal (grade 1 or 2) or by presence of one severe hypersignal lesion (grade 2 only), regardless of the presence of additional smaller lesions. The reliability of both scoring systems for meniscal and bone changes was previously demonstrated to be excellent [7].

### Statistical analysis

All of the data (clinical, radiological, and laboratory) were systematically entered into a computerized database using a blinded double-entry procedure, after which descriptive statistics for patient characteristics were tabulated. The cartilage volume losses are presented as percentage losses compared with baseline (mean ± standard deviation) and statistical relevance assessed by a one-sample Student *t *test. A set of analyses was done by dividing the cohort into quartiles of cartilage volume loss, the first quartile demonstrating greater cartilage volume loss. Statistical relevance was assessed using a Fisher exact test for categorical data and a two-sided Student *t *test for continuous data. The relationship between subregion cartilage volume loss at 24 months and the patient baseline characteristics, such as demographics, symptoms, JSW, and other MRI findings, was investigated using the Spearman correlation test. Finally, multivariate forward stepwise correlations were used to assess predictors of cartilage volume loss independently of potential confounders. All statistical analyses were done using Statistica, version 7 (StatSoft, Inc., Tulsa, OK, USA). All tests were two-sided, and a *p *value of 0.05 was considered statistically significant. Analyses were not corrected for multiple comparisons.

## Results

### Patient characteristics

A total of 107 out of 110 patients were assessed with qMRI; three patients were lost to follow-up. At baseline, the cohort demographics and characteristics were largely in line with the OA population of the main study [13]: the mean age was 62.4 ± 7.5 years, 64% of subjects were female, subjects had an average BMI of 30.6 ± 4.3 kg/m^2^, the duration of knee OA was 8.9 ± 7.2 years, 91.4% were taking analgesics and 72% were taking NSAIDs, and these patients were exhibiting disease activity scores in the mild to moderate range according to the WOMAC (total: 38.9 ± 22.9), the patient global (visual analog scale: 48.2 ± 5.0), the SF-36 (38.1 ± 9.5), and the Kellgren-Lawrence (grade 2: 53% of the patients; grade 3: 47%) scales. The mean JSW measurement at baseline was 2.88 ± 0.64 mm.

The patient cohort population characteristics at baseline were first analyzed by quartile, separating those with the greatest loss (first quartile) of global and medial cartilage volume from those with the least loss (fourth quartile). Data from global volume (Table 1) showed that the greatest risks for rapid progression from the demographic characteristics were the body weight (*p *< 0.07) and the BMI (*p *< 0.06). From a structural point of view, the most predominant risk factors were the presence of a severe medial meniscal tear (*p *< 0.01) or medial meniscal extrusion (*p *< 0.02) and the most statistically significant risk was associated with the severe medial meniscal extrusion (complete extrusion; *p *< 0.0001). Moreover, the presence of a bone marrow hypersignal in the lateral compartment was also associated with a greater risk (*p *< 0.005). For the medial compartment (Table 1), the greatest risks from the demographic characteristics were the female gender (*p *< 0.05), the BMI (*p *< 0.07), and the SF-36 (*p *< 0.07). Of note was the strongly predictive value of the JSW at baseline (*p *< 0.01). From the structural changes, the risk factors were similar to those found for the global volume with the addition of the medical meniscal tear (*p *< 0.02) and the bone hypersignal in the medial compartment (*p *< 0.06).

**Table 1:** Patient characteristics at baseline: first versus fourth quartile based on cartilage volume loss

	Global			Medial compartment		
	First quartile (greatest loss) (*n *= 27)	Fourth quartile (least loss) (*n *= 27)	*P *value	First quartile (greatest loss) (*n *= 27)	Fourth quartile (least loss) (*n *= 27)	*P *value

Age	63.7 ± 7.2	61.3 ± 7.5	0.23	64.1 ± 7.4	61.6 ± 7.8	0.24
Female	58%	67%	0.50	48%	74%	0.05
Weight (kg)	84.9 ± 14.9	77.6 ± 14.3	0.07	86.3 ± 14.9	78.6 ± 16.3	0.07
Body mass index	31.4 ± 3.9	29.1 ± 4.9	0.06	31.4 ± 3.6	29.6 ± 4.8	0.14
Joint space width (mm)	2.84 ± 0.54	3.06 ± 0.64	0.18	2.70 ± 0.67	3.18 ± 0.66	0.01
WOMAC pain	38.8 ± 22.1	35.4 ± 23.7	0.54	37.3 ± 23.2	34.1 ± 23.0	0.60
WOMAC function	44.3 ± 23.3	38.0 ± 24.8	0.34	43.6 ± 23.9	37.2 ± 24.8	0.33
SF-36	39.0 ± 8.8	34.8 ± 9.8	0.10	40.2 ± 8.3	35.9 ± 8.4	0.07
Severe medial meniscal tear	50%	19%	0.01	51%	11%	0.002
Medial meniscal tear	85%	81%	0.76	92%	66%	0.02
Severe medial meniscal extrusion	54%	7%	0.0001	56%	7%	0.0003
Medial meniscal extrusion	81%	52%	0.02	82%	48%	0.01
Bone hypersignal	77%	48%	0.03	81%	37%	0.002
Bone hypersignal in the medial compartment	62%	48%	0.34	59%	33%	0.06
Bone hypersignal in the lateral compartment	50%	15%	0.005	59%	15%	0.001

Values are mean ± standard deviation, *p *values are two-sided Student *t *test; or values are in percentage, p values are Fisher exact test. WOMAC, Western Ontario and McMaster Universities Osteoarthritis Index.

### Cartilage volume loss on condyles, plateaus, and subregions

The analysis of the cartilage volume/thickness loss in the different anatomical areas of the knee was most informative. The greatest percentage of cartilage volume loss over time (24 months) was found in the medial condyle and plateau, followed in order by the lateral plateau and the trochlear area (Figure 1; Table 2). Further analysis by subregion indicated that the greatest loss of cartilage volume on the condyles was occurring in the central (weight-bearing) and anterior portions of the medial condyle. At the trochlear level, the greatest loss was on the medial section. On the tibial plateaus, the maximum loss was found on the medial plateau and was approximately 35% greater than that found on the lateral plateau. Plateau subregion analysis revealed findings similar to those for the condyles. The maximum loss was found in the central (weight-bearing) portion of the medial plateau followed by the anterior and then the posterior section. On the lateral plateau, the maximum loss was also found, as for the medial plateau, on the central weight-bearing area. However, in contrast to the latter, a much greater loss was found on the posterior section than on the anterior section. Compared with baseline, each of the subregions, except for the anterior and posterior subregions of the lateral condyle and anterior subregion of the tibial plateau, was statistically different (*p *< 0.0001) (Table 2).

**Table 2:** Cartilage volume in absolute value (mm^3^) and change over 24 months follow-up from baseline

	Baseline (mm^3^)	24 months (mm^3^)	Change at 24 months (mm^3^)	Change at 24 months (percentage)
Femoral condyle				
Medial	2,228.9 ± 609.1	2,028.3 ± 585.1	-201.5 ± 178.6	-9.1 ± 7.5
Lateral	2,155.2 ± 677.6	2,105.8 ± 654.3	-43.8 ± 94.5	-2.0 ± 4.6
Trochlea	2,974.4 ± 901.4	2,835.7 ± 849.5	-123.8 ± 156.6	-4.1 ± 5.1
Transversal subregions				
Medial				
Posterior	840.6 ± 240.5	802.7 ± 236.5	-37.9 ± 70.2	-4.4 ± 7.9
Central	729.2 ± 240.3	643.3 ± 232.9	-87.9 ± 90.4	-12.0 ± 11.5
Anterior	659.2 ± 213.3	582.2 ± 212.0	-75.7 ± 72.8	-12.4 ± 12.0
Trochlea	1,307.6 ± 404.9	1,225.7 ± 365.0	-77.8 ± 90.9	-5.5 ± 6.1
Lateral				
Posterior	578.6 ± 192.9	571.9 ± 193.1	-4.8 ± 43.5	-0.9 ± 7.6
Central	931.9 ± 323.8	903.0 ± 312.2	-28.3 ± 53.4	-2.9 ± 6.2
Anterior	644.7 ± 210.2	631.0 ± 203.2	-10.8 ± 32.5	-1.7 ± 5.6
Trochlea	1,666.7 ± 537.8	1,610.1 ± 529.9	-46.0 ± 98.7	-3.0 ± 6.8
Tibial plateau				
Medial	1,320.4 ± 419.9	1,195.0 ± 373.2	-126.0 ± 117.0	-9.3 ± 7.5
Lateral	1,673.8 ± 520.8	1,563.1 ± 466.9	-101.9 ± 98.1	-6.1 ± 5.7
Concentric zones				
Medial				
Border ring	709.0 ± 242.0	660.1 ± 211.9	-47.9 ± 57.1	-6.2 ± 6.3
Center ring	611.4 ± 192.6	534.9 ± 181.8	-78.1 ± 70.0	-13.0 ± 10.9
Lateral				
Border ring	790.2 ± 249.1	746.3 ± 224.0	-39.0 ± 44.3	-5.0 ± 5.7
Center ring	883.6 ± 286.1	816.8 ± 260.3	-62.9 ± 60.3	-7.2 ± 7.8
Transversal subregions				
Medial				
Posterior	397.6 ± 140.5	380.1 ± 128.4	-17.1 ± 36.1	-3.7 ± 7.7
Central	566.7 ± 191.6	484.6 ± 179.7	-84.2 ± 72.4	-15.0 ± 12.0
Anterior	356.1 ± 130.2	330.3 ± 116.7	-24.7 ± 36.0	-6.8 ± 10.3
Lateral				
Posterior	515.5 ± 176.7	474.9 ± 154.5	-36.6 ± 41.3	-7.1 ± 8.9
Central	791.7 ± 265.4	729.7 ± 244.4	-59.5 ± 56.0	-7.8 ± 8.4
Anterior	366.5 ± 122.3	358.4 ± 115.2	-5.8 ± 18.3	-1.6 ± 5.0

The data are the mean ± standard deviation. The *p *values from one sample two-sided Student *t *test in the change of cartilage volume are less than 0.0001 for all regions except for the lateral femoral condyle at the anterior and posterior areas and for the lateral tibial plateau at the anterior area.

### Correlations between cartilage volume loss at 24 months in the central areas of the medial compartment and the demographic, clinical, structural, and joint space width data

The analyses focused first on MRI data from the central areas of the medial compartment as they presented the greatest loss of cartilage volume and were therefore the areas of the most significance (Table 3). From the baseline demographic characteristics, a positive and significant correlation was found with the BMI at the regions of interest, which included the central femoral condyle and plateau and the two subregions combined together (compartment). With regard to the structural changes, correlations were obtained for the cartilage loss in the central area of the medial femur and tibia and both subregions combined, compared with JSW, the presence of meniscal extrusion and severe meniscal extrusion, severe medial meniscal tear, and the subchondral bone marrow hypersignal, particularly in the lateral compartment. There was also a correlation between the loss of cartilage in the medial central femoral condyle and the presence of lateral meniscal tear. For JSW at baseline, a trend was found between alcohol consumption and statistical significance with medial meniscal tear and severe medial meniscal tear. With regard to the changes in clinical variables at 24 months and the loss of cartilage, data revealed significant correlations between the clinical criteria WOMAC pain score change at the plateau and medial compartment levels. There was also a trend between the patient global change and the medial compartment. For the x-rays, a significant correlation was obtained between the joint space narrowing (JSN) and the WOMAC total as well as with the WOMAC pain and function subscales. The loss in JSW (JSN) was significantly correlated with the loss of cartilage volume on the central weight-bearing area of the condyles and the plateaus as well as on the medial compartment.

**Table 3:** Univariate Spearman correlations with cartilage loss at 24 months

Baseline characteristics	Medial central femoral condyle		Medial central tibial plateau		Both areas (medial compartment)		Joint space width (mm)	
		*P *value		*P *value		*P *value		*P *value

Age	-0.13	0.18	-0.07	0.49	-0.11	0.26	-0.05	0.58
Gender	0.12	0.24	0.08	0.41	0.11	0.26	0.04	0.66
Body mass index	-0.21	0.03	-0.21	0.03	-0.23	0.02	-0.07	0.48
Alcohol	0.02	0.86	0.02	0.82	0.02	0.82	0.18	0.06
Smoking	0.74	0.45	0.09	0.36	0.09	0.35	0.06	0.55
SF-36	-0.14	0.14	-0.03	0.79	-0.09	0.34	-0.05	0.61
WOMAC total	-0.05	0.61	-0.02	0.82	0.02	0.88	0.08	0.41
WOMAC subscale								
Pain	0.10	0.30	0.05	0.58	0.09	0.38	0.14	0.14
Stiffness	0.04	0.68	-0.07	0.46	-0.06	0.52	-0.0005	0.99
Function	-0.05	0.62	-0.03	0.76	0.01	0.92	0.08	0.42
Patient global	0.04	0.65	0.12	0.90	0.03	0.75	-0.0004	0.99
Joint space width (mm)	0.29	0.003	-0.28	0.03	0.32	0.001	--	--
Meniscal extrusion	-0.31	0.001	-0.26	0.007	-0.32	0.001	-0.16	0.11
Severe meniscal extrusion	-0.33	0.001	-0.40	0.0001	-0.41	0.0001	-0.16	0.10
Medial meniscal tear	-0.26	0.006	-0.11	0.25	-0.21	0.03	-0.32	0.001
Severe medial meniscal tear	-0.36	0.0001	-0.29	0.003	-0.36	0.0001	-0.32	0.001
Lateral meniscal tear	0.21	0.03	0.05	0.60	0.15	0.13	-0.09	0.33
Severe lateral meniscal tear	0.07	0.49	0.04	0.65	0.06	0.52	0.16	0.11
Bone hypersignal	-0.26	0.008	-0.23	0.02	-0.27	0.005	-0.11	0.25
Bone hypersignal in the medial compartment	-0.11	0.23	-0.10	0.30	-0.12	0.21	-0.03	0.76
Bone hypersignal in the lateral compartment	-0.32	0.001	-0.31	0.001	-0.35	0.0001	-0.13	0.17
								
Changes in selected clinical variables at 24 months	Medial central femoral condyle		Medial central tibial plateau		Both areas (medial compartment)		Joint space narrowing	

		*P *value		*P *value		*P *value		*P *value

SF-36	0.07	0.44	0.12	0.23	0.11	0.27	0.10	0.31
WOMAC total	-0.05	0.58	-0.10	0.31	-0.09	0.38	-0.23	0.02
WOMAC subscale								
Pain	-0.15	0.12	-0.21	0.03	-0.21	0.03	-0.29	0.002
Stiffness	0.02	0.85	-0.03	0.74	0.009	0.93	-0.18	0.07
Function	-0.04	0.68	-0.07	0.45	-0.06	0.51	-0.23	0.02
Patient global	-0.17	0.08	-0.17	0.08	-0.19	0.05	-0.02	0.83
Joint space narrowing	0.40	0.003	0.21	0.003	0.34	<0.0001	--	--

WOMAC, Western Ontario and McMaster Universities Osteoarthritis Index.

### Forward stepwise multivariate correlations

The most statistically significant independent predictors of the loss of cartilage volume in the central area of the medial femoral condyles (Table 4) at baseline were (in order of significance) the loss of joint space (JSN), the presence of a lateral meniscal tear, the SF-36 score, the BMI, and to a lesser extent the JSW. Interestingly, the most significant independent predictors of the loss of cartilage volume in the central area of the medial tibial plateaus (Table 5) were to some extent different from those found for the femoral condyle. These include (in order of significance) the presence of a severe meniscal extrusion, an increase in the WOMAC pain score, the presence of bone hypersignal and severe meniscal tear in the lateral compartment, and to a lesser extent the WOMAC stiffness.

**Table 4:** Parameters predicting medial central femur cartilage volume loss at 24 months: stepwise forward multivariate regression

	Beta coefficient	Standard error of beta	*P *value
Joint space narrowing	0.29	0.08	0.0005
Lateral meniscal tear	0.24	0.08	0.005
SF-36	-0.22	0.09	0.02
Body mass index	-0.19	0.09	0.03
Joint space width (mm)	0.18	0.10	0.06
Alcohol	-0.15	0.08	0.07

Correlations were done with baseline values. Values were adjusted by factors listed in Table 3.

**Table 5:** Parameters predicting medial central tibia cartilage volume loss at 24 months: stepwise forward multivariate regression

	Beta coefficient	Standard error of beta	*P *value
Severe meniscal extrusion	-0.33	0.09	0.0004
WOMAC pain (change)	-0.26	0.09	0.007
Bone hypersignal in the lateral compartment	-0.19	0.09	0.03
Severe lateral meniscal tear	0.21	0.09	0.02
WOMAC stiffness	-0.16	0.09	0.07

Correlations were done with baseline values unless otherwise indicated. Values were adjusted by factors listed in Table 3. WOMAC, Western Ontario and McMaster Universities Osteoarthritis Index.

The risk factors associated with the loss of cartilage volume in the central subregions of the medial compartment (combination of femoral condyle and tibial plateau) (Table 6) were (in order of significance) the presence of a severe meniscal extrusion, the JSN, the presence of a bone hypersignal in the lateral compartment, and alcohol consumption.

**Table 6:** Parameters predicting the medial central subregion^a ^cartilage volume loss at 24 months: stepwise forward multivariate regression

	Beta coefficient	Standard error of beta	*P *value
Severe meniscal extrusion	-0.28	0.10	0.004
Joint space narrowing	0.18	0.09	0.03
Bone hypersignal in the lateral compartment	-0.19	0.09	0.04
Alcohol	-0.17	0.09	0.04
Severe medial meniscal tear	-0.16	0.09	0.08
Severe lateral meniscal tear	0.15	0.09	0.08
Body mass index	-0.15	0.09	0.09

^a^Medial central femur and tibia combined. Correlations were done with baseline values. Values were adjusted by factors listed in Table 3.

## Discussion

This longitudinal study provides new and interesting information about the risk factors associated with the rapid advancement of the disease progression (cartilage loss) in patients with symptomatic OA. It also brings to light new and unique information about the topographical loss of cartilage in the different subregions of the knee and the associated risk factors. Moreover, the impact of the location and rate of cartilage loss on the evolution of OA symptoms over time was thoroughly explored. Treatment with risedronate did not interfere with the actual results of the study as the drug was shown to have no significant effect on the loss of cartilage volume [7], on other structural changes, or on the disease symptoms or JSW changes over time [7,13].

The global (continuous) analysis of these data was previously done and provides very informative findings [7]. However, to further explore the risk factors that are selectively associated with more rapid disease progression, we performed analysis of cartilage volume loss by quartile in which we segregated the first quartile (greatest loss) from the fourth (least loss). Patients from the first quartile are of particular interest from a clinical perspective as they are likely to have the worst prognosis and are therefore at greater risk of surgical intervention for joint replacement. Moreover, they are of special interest for DMOAD studies as they may be the most likely to respond to treatment [27].

The results of this study with regard to the quartile analysis show that OA patients experiencing the greatest risk of cartilage loss (first quartile), and more particularly in the medial compartment, were female and had a higher BMI. There was, however, no difference at baseline in disease symptoms or patient function. Interestingly, these patients showed a significantly narrower JSW. From a structural point of view, severe medial meniscal tear and/or extrusion or subchondral bone marrow hypersignal predominantly in the lateral compartment were clearly the most significant risk factors (Table 1). These are in line with some of our findings from previous analyses [2,7] and with the work of other investigators [9,11,28,29]. There are, however, some exceptions. For instance, the association of the extent of cartilage loss with disease symptoms and, more precisely, with the WOMAC and SF-36 scores was lost, although a trend was found for the SF-36. We believe that this may be due to a type II error given that the groups of patients in the present analysis were relatively small. However, other explanations are also possible (for example, the cross-sectional nature of this type of analysis which also imposes limitations on our results).

These results are of great interest and have practical implications as they show that factors that can predict disease progression can be identified at the time that patients are included in clinical studies, as previously mentioned. These findings may also have particular relevance to DMOAD trials, as the selection of patients who present little or no progression of structural changes, specifically cartilage loss over time, represents a major challenge. This is particularly true with respect to the calculation of the number of patients to be included in a clinical trial. It also raises the important issue of the relevance of performing patient stratification at baseline during clinical trials.

Our findings on the key role played by the meniscus in OA cartilage pathology and loss nicely complement a number of previous reports. For example, MRI studies that have examined the role of meniscal lesions in OA and non-OA populations have reported that these changes [28,30] are correlated with a greater risk of cartilage loss [9]. The findings of this study are supported by both univariate and multivariate analyses. Similar findings were reported with meniscal malposition in patients with symptomatic knee OA [9] and in patients who had undergone partial meniscectomy [31]. Taken together, these findings stress the very important role played by the meniscal structure and positioning in protecting the integrity of cartilage in both healthy and OA individuals. Our data, however, clearly point to the fact that by far the greatest risk was associated with the presence of a complete (severe) extrusion of the medial meniscus. These findings support the hypothesis that the meniscus exerts a direct protective effect by reducing the physical contact between the cartilage surfaces. They also point to a protective effect of the meniscus even in the presence of degenerative changes as long as it remains in its normal positioning.

The present study showed that bone marrow hypersignal was also a risk factor for rapid loss of cartilage for the global knee and in the medial compartment. Interestingly, it was the presence of a bone hypersignal in the lateral compartment that was found to be the most significant risk factor. The relationship between bone marrow hypersignal and severity of knee OA cartilage lesions was first shown by Hunter and colleagues [29] and Felson and colleagues [32]. The compartmental edema was correlated with local cartilage loss as well as with limb alignment (medial lesions being associated with varus limbs and lateral lesions with valgus limbs). In the present study, any patients with clinically significant malalignment were excluded. However, although an examination was performed, there was no precise measurement of joint alignment. Thus, no firm conclusion can be arrived at as to the role played by malalignment in the present study. The exact reasons for the relationship between the presence of bone marrow hypersignal in the lateral compartment and the rapid loss of cartilage in the medial compartment remain unexplained and require further exploration. Though speculative, one possible explanation could be that patients experiencing such damage tend to shift their weight, putting more biomechanical stress on the lateral compartment.

The evaluation of cartilage loss by means of subregional analysis provides most interesting information. The loss was much greater in the medial compartment for both femoral condyles and tibial plateaus. This sensitivity to change being greater in the medial compartment was somewhat expected as patients selected for this study had baseline OA that was predominant in this particular compartment. These data support the validity of such outcome measures. On the femoral condyle, the loss was found in the central and anterior sections, followed by the trochlear area. These findings are in line with the work of Amin and colleagues [4] in patients with knee OA with meniscal lesions and that of Biswal and colleagues [30] in patients with meniscal tears who had undergone meniscectomies, with the exception that the preferential loss of cartilage occurring at the posterior medial femoral condyle found in these two studies was not confirmed in our OA population. The loss of cartilage on the lateral condyle followed the same pattern as the medial condyle but with the loss being less pronounced. The loss of cartilage on the tibial plateaus was identified mainly in the central area, which is not covered by the menisci. In addition, the pattern of loss in transversal subregions was found to be different between the two plateaus; in the medial plateau a greater loss was found in the anterior area, whereas on the lateral plateau it was on the posterior area. These differences point to the possibility that risk factors leading to cartilage loss could have been different in each tibial plateau. The low incidence of meniscal lesions in the lateral compartment [2] points to the likelihood that other factors, such as bone marrow hypersignal, may play a more predominant role at that level, as indicated by the results from the quartile analysis. However, on the medial side, the preferential loss of cartilage in the central and anterior areas correlates closely with the high prevalence of tear/extrusion at that level, since no posterior lesions could be found in these patients [2].

Comparative data from the MRI and x-rays indicate that the MRI is a more comprehensive tool for globally identifying factors that are predictive of the progression of the disease. Both the JSN and the cartilage volume loss were found to be positively correlated with the worsening of pain. The cartilage loss also correlates with the patient global score, and JSN with the loss of function. However, there was not good concordance between the two methods, with the exception of the WOMAC pain. Again, this may be due to the limitations imposed by the univariate analysis.

This study also provides clear evidence of a correlation between the loss of cartilage on weight-bearing areas and OA disease symptoms. The predictive value of the SF-36 indicates that the patients at high risk of progression are usually more disabled by the disease. They also experienced an increase in the level of knee pain, and, to a lesser extent, joint stiffness over time as the disease progressed. The relationship between the worsening in the WOMAC scores with the loss of cartilage on the central portion of the tibial plateau is intriguing. The level of significance of these findings for the WOMAC pain is greater than those previously reported for the loss of cartilage volume in the entire medial compartment [7]. Moreover, the trend toward correlation with joint stiffness is a new and most interesting finding, as there is, to date, very little information of this kind originating from longitudinal studies. However, from cross-sectional studies, there have been reports of the correlation of knee pain with full-thickness cartilage defects [3,6] and bone marrow lesions [3,6,32]. Such a study discriminating subregions is of great significance in DMOAD trials as the effect of drug treatment on structural changes needs to be correlated with disease symptoms.

Our findings provide new information about the possible correlation between the loss of cartilage volume assessed by MRI and the loss of JSW assessed by x-ray. Previous studies conducted by our group [7,22] demonstrated the absence of correlation between the loss of cartilage volume in the entire medial compartment and the JSW in patients with knee OA. These findings were in line with the previous report of Amin and colleagues [4]. However, in the present study, a very strong correlation was found between the JSN and the loss of cartilage in the central area of the medial femoral condyle and, to a lesser extent, with the loss on the medial central tibial plateau. These findings are in close accordance with patient knee positioning during x-ray exams. Therefore, the standard x-ray technique and consequently JSW measurement commonly used in clinical trials accurately assess the focal loss of cartilage in patients with knee OA in the medial condyle and plateau subregions. Compared with x-rays, MRI presents significant advantages in assessing the change in cartilage volume/thickness in all the other subregions and compartments of the knee in addition to providing information on other structural changes, including the meniscus and subchondral bone. The latter are most relevant in identifying predictive factors of disease progression. Moreover, the evaluation of changes in both medial and lateral compartments is imperative given that a recent study demonstrated that some drugs may have a preferential chondroprotective effect on the loss of cartilage in the lateral compartment [27].

This study has obvious limitations, the main one being the relatively small number of patients included in the analyses. New studies are under way or have been recently completed [27] and should provide additional information and hopefully confirm the present findings. Another limitation is the fact that some patients received treatment with risedronate. All efforts have been made to ensure that this had no impact on the actual findings; the published data from the entire patient cohort analysis are certainly supportive to that effect [13].

## Conclusion

This study provides new information on the risk factors associated with the loss of cartilage and the evolution of symptoms in patients with knee OA in which meniscal damage and bone marrow changes are the features most closely associated with subregional cartilage loss. JSW narrowing was demonstrated to be strongly associated with cartilage loss in weight-bearing areas. This reflects that JSW change at its narrowest point may be closely related to cartilage loss in specific subregions. Lastly, these data also confirm the correlation between cartilage volume loss and the severity of symptoms, including worsening of pain at 24 months.

## Abbreviations

BMI = body mass index; DMOAD = disease-modifying osteoarthritis drug; FISP = fast inflow with steady-state precession; JSN = joint space narrowing; JSW = joint space width; MRI = magnetic resonance imaging; NSAID = nonsteroidal anti-inflammatory drug; OA = osteoarthritis; qMRI = quantitative magnetic resonance imaging; WOMAC = Western Ontario and McMaster Universities Osteoarthritis Index.

## Competing interests

JFB, GAC, and JMM declare that they have financial competing interests as employees of and/or holders of stocks and/or options in Procter & Gamble Pharmaceuticals (Mason, OH, USA). FA declares that he has financial competing interests as an employee of and/or holder of stocks and/or options in ArthroVision (Montreal, QC, Canada). J-PP and JM-P declare nonfinancial competing interests as consultants of Procter & Gamble Pharmaceuticals and financial competing interests and/or holder of stocks and/or options in ArthroVision. M-JB and J-PR declare nonfinancial competing interests as consultants of ArthroVision.

## Authors' contributions

J-PP and JM-P contributed to study design, acquisition of data, analysis and interpretation of data, manuscript preparation, and statistical analysis. J-PR contributed to study design, acquisition of data, analysis and interpretation of data, and statistical analysis. M-JB and FA contributed to acquisition of data and to analysis and interpretation of data. DC and BH contributed to acquisition of data. JFB, GAC, and JMM contributed to manuscript preparation. All authors read and approved the final manuscript.
